# An ecological systems approach to the difficulties faced by social workers in community home-based care organisations for people living with HIV

**DOI:** 10.1080/21642850.2023.2236181

**Published:** 2023-07-18

**Authors:** Maditobane Robert Lekganyane, Tsakani Salphinah Manganyi

**Affiliations:** a Department of Social Work, University of South Africa, Pretoria, South Africa; b Zanempilo Home Based Care, Pretoria, South Africa

**Keywords:** Challenges, social worker, HIV

## Abstract

**Background:**

Social workers play an instrumental role in supporting vulnerable populations by among others, designing and implementing support programmes such as community home-based care (CHBC) for People Living with HIV (PLWHIV). Existing research studies have demonstrated their role in championing programmes like support groups, counselling services and material support to PLWHIV and their caregivers as well as the HIV prevention and other advocacy programmes across communities. Within the CHBC programmes for HIV, social workers’ roles include supporting caregivers who care for PLWHIV to manage complex cases that are beyond their competencies and offering the necessary training on patient support. Although the contributions made by social workers in the field of HIV have immensely been documented in various parts of literature, less substantial attention has been given to their challenges particularly when working with PLWHIV through CHBC programmes.

**Methods:**

This qualitative research study was designed from exploratory and descriptive strategies and grounded on ecological systems theory to explore the challenges faced by social workers working with PLWHIV through the CHBC programmes. Thirteen social workers were sampled from South Africa’s City of Tshwane Municipality (CTMM) through purposive and snowball techniques, to participate in semi-structured interviews of which the data were analysed thematically and verified according to Lincoln and Guba’s data qualitative data verification strategies.

**Findings:**

The findings highlighted several difficulties faced by social workers including difficulties associated with managing challenges faced by PLWHIV, dealing with uncooperative PLWHIV and lack of resources to effectively respond to their clients’ needs.

**Conclusions:**

The conditions in which social workers render services to PLWHIV were highlighted by pointing to the complexities resulting from these challenges which further compromises the quality of services rendered to PLWHIV. For the success of HIV programmes, it is essential to support social workers through training, resources, community awareness and income-generating projects.

## Introduction

The medical advancement in the field of Human Immunodeficiency virus (HIV) and Acquired Immune Deficiency Syndrome (AIDS) has turned AIDS into a chronic condition, necessitating continuous care, treatment and support from professionals such as social workers. Initiatives like CHBC were introduced to provide health care and social support services within the patients’ households (Van Dyk, 2012, p. 344). Through CHBC programmes, social workers play a crucial role in supporting PLWHIV to manage emotional distress and related psychosocial conditions (Ntshwarang & Malinga-Musamba, 2012, p. 291). Community home-based care services seek to create hope, best quality of life and independence to patients and members of their families (Larki & Roudsari, 2019, p. 190). These services include nursing care, palliative care and rehabilitative care (Campbell et al., 2019, p. 3; Larki & Roudsari, 2019, p. 191). *Nursing care* provides basic nursing care and comforting services such as symptom management, patient diagnosis and treatment, positioning, moving and bathing patients, cleaning and dressing wounds, oral hygiene, ventilation guidance and nutritional support, as well as referrals (Mamukeyani, 2021, p. 2). *Palliative care* addresses the physical, psychological, emotional, social and spiritual needs of the individual and family with sensitivity to their personal, cultural and religious values, beliefs and practices (Moremi, 2012). With *rehabilitative care*, mentally and physically disabled patients are guided on using devices like crutches and wheelchairs, and family members are educated on managing a sick member and his/her condition (Lekganyane, 2017, p. 6). Caregiving is an invisible economy embracing caring for children, the elderly and the sick and it is largely driven by unpaid and unrecognised women (Viphonephom et al., 2021, p. 116).

In South Africa, the CHBC takes the form of integrated model, a single service model and an informal model (Larki & Roudsari, 2019, p. 194; Van Dyk, 2012, p. 346). An *integrated CHBC model* provides patients with various services by trained caregivers who are supported and supervised by healthcare professionals and in partnerships with support groups, non-governmental organisations (NGOs), community-based organisations (CBOs) and the entire community (Mamukeyani, 2021, p. 7; Van Dyk, 2012, p. 346). In *single service CHBC model*, the clinics, hospitals, NGOs or churches recruit and train volunteers and link them with patients and their families at home so that they can care for and support these patients (Campbell et al., 2019, p. 3; Van Dyk, 2012, p. 3 247). *Informal HBC involves families* caring for sick members at home with the assistance of extended family and the community (Campbell et al., 2019, p. 7; Larki & Roudsari, 2019, p. 194). Common forms of CHBC model are the integrated model and the single service model wherein volunteers are recruited, trained and organised to render CHBC services (Van Dyk, 2012, p. 346).

Community home-based caregivers work in most difficult conditions characterised by poverty, hopelessness and demotivation resulting from lack of recognition of their work (Kupa & Geyer, 2020, p. 2). They work in stressful, depressive and frustrating conditions (Pindani et al., 2013, p. 2013:4; Viphonephom et al., 2021). A Malawian study by Pindani et al. (2013, p. 4) for example revealed negative attitudes by community members towards PLWHIV as a result of lack of knowledge. Caregivers who took part in this study reported being ill-informed and ill-prepared when rendering services (Pindani et al., 2013, p. 4). In Viphonephom et al. (2021), researchers devoted time to examine how and why caregivers of PLWHIV faced difficulties and found that they were burdened by work which in turn led to multiple stresses such as illness management, HIV stigma and its related social impact, communication with healthcare providers and social service agencies and experiencing multiple AIDS-related deaths. In further complicating the caregivers’ stressors was a lack of material, emotional and financial support for children living with HIV and their caregivers from families and communities (Viphonephom et al., 2021, p. 118). This places social workers at the centre of CHBC because their extensive training and scope of practice give them a distinguished ability to assess the psychosocial needs of the patients and caregivers and the necessary collaborative treatment plans (Reckrey et al., 2014, p. 6). Social workers can therefore relieve these caregivers from exhaustive and stressful conditions.

Given the entrenched nature of social work in CHBC, it is generally impossible to conclude any discussion of this field without advancing their role. In South Africa, HIV programmes are rolled down across three spheres of government. The national government which is mainly responsible for designing national strategies and policies, the provincial government which is responsible for overseeing the implementation of national strategies and policies in their respective provinces and the local government which in partnerships with NGOs and CBOs such as the CHBC, is responsible for implementing the policies and strategies. The NGOs and CBOs provide various services, with some such as the CHBC specialising in HIV services. Social workers serve in all spheres of government as well as in NGOs and CBOs like CHBC to specifically render support to PLWHIV and their caregivers. In the CHBC, they provide counseling and support PLWHIV towards treatment adherence and liaise with various stakeholders involved in the patient’s life by facilitating preservation services, educating patients and families, and mobilising resources for the benefit of patients and families (Freeman, 2017, p. 106; Reckrey et al., 2014, p. 4; Verma, 2020, p. 2). CHBC services offered by social workers also extend to caregivers themselves through debriefing, support groups and liaising with health care professionals for resources and professional expertise (Lekganyane & Alpaslan, 2019, p. 142; Mamukeyani, 2021, p. 12; Valjee & Van Dyk, 2014, p. 421; Qalinge, 2011, p. 54). In some instances, social workers relieve the caregiver’s burden by managing complex patient’s socio-economic problems, such as the application and processing of pension funds and disability grants (Bester & Herbst, 2010, p. 453; Kupa & Geyer, 2020, p. 8; Mamukeyani, 2021).

Despite the crucial role of social workers in CHBC for HIV, there is generally scanty literature hence this study sought to explore the challenges faced by social workers working with PLWHIV in CHBC. Following the Ecological Systems Theory (EST), it was anticipated that this study will enhance the practice, training and education of caregivers, social workers and related professional by providing the empirical evidence upon which intervention strategies can be designed and developed. The EST is traced back from the 1979 work of Bronfenbrenner who believed that an individual’s life develops around the *microsystem*, the mesosystem, the exosystem and the macrosystem (Mahoney & Ettekal, 2017, p. 2; Navarro & Tudge, 2022, p. 18–719; White & Hayes, 2013, p. 16). In the microsystem a person’s life develops from one immediate environment, like workplace, school or home environment (Crawford, 2020, p. 1; Drakenberg & Malmgren, 2013, p. 119). The microsystem in this context is the CHBC organisation where social workers render services to PLWHIV and PLWHIV are developing persons at the centre. In the *mesosystem*, two systems mutually interact to influence the individual’s development (Crawford, 2020, p. 2; Drakenberg & Malmgren, 2013, p. 119; Navarro & Tudge, 2022, p. 719) and it is where CHBC organisations interact with funding organisations and other referral networks to support social worker’s efforts.

The *exosystem* is wherein the developing person (PLWHIV) is not actively participating. It is where factors or events affect events in other settings (Crawford, 2020, p. 2; Drakenberg & Malmgren, 2013, p. 119). Here various factors affect developments in CHBC organisations without directly affecting the PLWHIV persons (Drakenberg & Malmgren, 2013, p. 119). Availability of resources or lack thereof from organisations to which patients are referred for further services might for instance impact on events that may unfold from CHBC organisations. Finally, the *macrosystem* is the wider established cultural or subcultural patterns influencing the person’s development (Navarro & Tudge, 2022, p. 719). The macrosystem is where one finds the deeply entrenched historical, structural, political and economic factors imposed on responses to HIV and seeking to undermine these responses by exacerbating the conditions in which they find themselves. Farmer (1999) points to factors such as poverty, inequality and cultural practices that facilitate the risk and impact of HIV in the Haitian context, which are common to most African countries (including South Africa). As argued by Farmer (1999, p. xxv), the risk of being infected with HIV for instance doesn’t depend on whether or not one knows how it is transmitted, but by her freedom to make decisions which is in turn limited by poverty. Similarly, the success of initiatives by social workers and caregivers in responding to HIV will be determined by factors such as poverty, inequality, political will which in turn availability or lack of resources and overall support. Any researcher seeking to understand the dimensions of this field should therefore be considerate of this array of factors.

## The research methods

This was an exploratory qualitative study conducted in South Africa’s Gauteng province, within the CTMM and designed from exploratory and descriptive research strategies. The population was social workers who were recruited from four CHBC organisations located in the CTMM. Purposive and snowball sampling were used to select the Participants who had to (1) have more than one year’s experience in rendering social work services to PLWHIV through an HBC organisation, (2) had to be willing to participate in the study out of their own volition, (3) be registered with the SACSSP as social workers, (4) either be employed on a full-time basis by these HBC organisations or serve in their voluntary capacity. From this criteria, twelve social workers were identified and recruited to participate. Using semi-structured individual face-to-face interviews which was aided by an interview guide with containing 6-item open-ended questions, researchers required Participants to share their experiences regarding, their responsibilities as social workers in the HIV CHBC; their challenges when directly working with PLWHIV; the challenges associated with their organisations; the strategies used in coping with these challenges and their suggestions on how to address the challenges. The interviews which lasted for approximately 60–90 min, were conducted and recorded in local languages by one of the researchers who was under training and guidance of an experienced supervisor who is well vested in qualitative research. The collected data were analysed using Braun and Clarke’s thematic analysis and quality assured through Guba and Lincoln’s trustworthiness criteria of credibility, transferability, confirmability and dependability.

## Ethical considerations

In observing the research ethical protocols, a research proposal was compiled and subjected to the institutional review committee for scrutiny and approval while relevant authorisation was sought from organisations under which these social workers were serving as well as from social workers themselves. All Participants gave written informed consent to participate while confidentiality, anonymity and information management were observed throughout the study. Personal details of the Participants were not captured, with pseudonyms adopted to substitute the real names of the Participants.

## Results

The results of this study are presented according to the demographical profiles of the Participants as well as the actual themes and subthemes that emerged from data analysis. Demographically, Participants were twelve social workers rendering social work services to PLWHIV through various HBC programmes either in their capacities as employees or as volunteers. All Participants were recruited from HBC organisations in the City of Tshwane Metro Municipality (South Africa’s capital city), with their ages ranging from 27 and 47. Of the twelve, ten were females and only two were males. Their total caseload ranged between 40 and 135. Table 1 below outlines the prevalent themes, their frequencies and supporting quotations.

**Table 1.** Themes, subthemes and supporting quotations.

Themes	Subtheme	Quotations
*Theme 1: Managing the psychosocial difficulties faced by clients*	*Subtheme1.1. non/or prolonged disclosure of a HIV-positive status by the clients*	*Some of the clients fear to disclose their status, especially to the new partner. Yes, they say that they are scared to be rejected by their partners. It’s becomes a challenge, because the clients will spread the infection … Yes, I’m concerned, because other partners don’t know anything about the health status of another partner … [Participant #1]*
		*Some of our clients, they don’t want family members to know about their status. I feel frustrated when the clients delay to disclosure of their own status to the family members. Yes, when you need family member’s support, you don’t know where to start … . [Participant #2]*
		*Some of the clients delay disclosing the status to their partner, because they scared to be rejected. Some of the clients say that if they disclose the status to the partner, they will blame them that is the one who comewith disease. It becomes difficult to do a home visit. Because another partner wants to know what we are doing. It is distressing to do a home visit, because I have to keep confidentiality and because I can’t disclose the client status without consent.* I *keep confidentiality until the client is ready to disclose their status. [Participant #3]*
		*Lack of clients to disclosure from their family members is challenging*. *It’s hard to collect documents from their family member. It delays me in terms of opening files, because when I ask for the documents, they will ask me, what you want to do with the document. I feel distressed when I’m unable to open file because of [a] lack of the document. [Participant #4]*
	*Subtheme 1.2. Denial of a HIV-positive status by the clients they accept it.*	*Yes, because it seems like they are in a stage of denial. I feel disappointed; however, I need to respect their choices, because it seems* that *the client has not accepted their status. I have to accept, because clients have their own values and belief. [Participant #5]*
		*We sometimes find clients that are in denial after testing HIV. It becomes hard for us to refer them for other services, such as support groups. Yes, because we need the client’s consent for him/her to be part of the support group. As a social worker, I have to give the clients time to adjust themselves. [Participant #3]*
		*It becomes a challenge to help some of the clients who are in denial, for instance, they aren’t interested in our services, because they believe that they are HIV-negative. Some of the clients tested positive, but they continue saying that they are HIV-negative. The clients have the right. There is nothing that I can do until they accept it. [Participant #6]*
		‘*Because it is difficult to assist clients and the family members more especial they are reluctant to accept that indeed the person is sick. It becomes a challenge most of the time to be engage on therapy session with someone who is reluctant to deal with experience most of the case whereby the clients are HIV-positive. Some of them are difficult to manage and there is nothing we can do. As a social worker, we should treat clients with respect and allow the clients self-determination regardless the situation.'* **[Participant #8]**
		‘*Eish* [echoing frustration], *to work with clients who are in denial of their own status is a problem*. *As a social worker working with PLWHIV, it is so painful to see clients who are denial about their own status, because it’s difficult to help clients who are in denial*. *It becomes a challenge when clients are in denial, because we are unable to provide them with counselling. I feel hopeless to do counselling; however, I put aside my frame of reference. However, I understand that every client is unique.'* **[Participant #12]**
		‘*Some of the clients even they tested positive, they don’t accept that they are positive. Some of them they say that it’s a calling from the ancestors. It is difficult as a social worker to help the client who is in denial. When the client is in denial, can’t pay attention when you provide with counselling. It’s disappointing to see clients deny their own status; however, every client is unique, because you are unable to render services, I give the clients time to improve themselves.'* **[Participant #13]**
		‘*Some of the clients, they don’t accept that they are HIV-positive. Some of the clients who come with referral when you ask about the status, they said they are HIV-negative. It becomes a challenge, because you are unable to help them when they are in denial. Yes, because our programme deals with HIV, so if they don’t disclose status to us, it is hard to assist them.'* **[Participant #4]**
		‘*Some of the clients even if they are tested positive, they are in denial of their own status. Some clients say that they are not positive test kit made mistake. It’s challenging to seeing clients in denial. It hurts when clients don’t believe on their own health; however, I need to respect the client’s right.'* **[Participant 9]**
	*Subtheme 1.3. Dealing with clients who do not have family support*	*I think the most common challenge I encounter is when clients don’t have support from their family members. F*or *example, one of my clients is being rejected by their families after they disclosed their status. It becomes a challenge, because PLWHIV need support, support from the family members encourage the clients to accept the HIV status. [Participant #11]*
		*I really feel hopeless to see some of the family members stigmatising their own member instead to give them support so that they will be able to deal with illness. Yes, because I believe as family members support each other as regardless the situation. [Participant #8]*
		*Mostly you find that other families are not supportive to the client*. *Some families, they neglect the client after they know about his/her status. It hurt to see clients suffering alone, because you will become worried about the client. Some of the clients who are unable to do anything, we refer them to hospice. We also refer some of the clients to another organisation who will not able to help. [Participant #9]*
		*It becomes a challenge when you realise that the some of the family members, they are reluctant to care of the clients. Yes, sometimes is difficult to help the clients without support of the family members. Hmmm, some clients’ needs support from the family members, for instance, that clients can get very ill during the night, so family members needs to support the clients. It frustrating when the family members not supporting each other. [Participant #10]*
		‘*It’s become a challenge, because some of the clients, their family members do not want to help them financially. It affects me emotionally, seeing clients and their family members facing poverty*. *Some of the clients, we refer them to other organisation to get food parcels, while other, we refer them to the community skills programme to empower themselves.'* **[Participant #4]**
*Theme 2: Managing uncooperative clients*	*Subtheme 2.1. Managing client s who refuse counselling*	*Some of them, they say that they don’t need counselling, they are fine [and yet] you will see that client needs help, but she refused it. It becomes a challenge to assist clients who are not ready to talk about their own status … [Participant #11]*
		*Some of the newly diagnosed clients reject counselling during the session. [Participant #9]*
		‘*When I have a session with the client and the client don’t come to the office.*’ **[Participant #5]**
		‘*It became a challenge when the clients do not attend. Some say that they have other things to do at home, while others are complaining about the language barrier. It is frustrating to run a support group, because things don’t always go per plan. You will plan to have a session with the group members and you will find that some of them are not coming*. *Nothing much can be done, because the support group is voluntary. Clients cannot be forced to participate.’* **[Participant #13]**
	*Subtheme 2.2: Poor uptake of referrals by the clients*	*Yes, because you find that the resources that they need, we don’t offer at our organisation. We refer them to get assistance elsewhere. It becomes a challenge when you refer the clients to other organisation to access the services. Some of the clients, they become reluctant when you refer them to another organisation … . Some of them are very difficult. For instance, you can refer them to skill programme, but they are unwilling to go. It becomes a challenge when the clients don’t go to improve themselves, while you take your effort to assist them. [Participant #10]*
		*Some of the clients, when you refer them to other stakeholder, they become reluctant to receive other services*. S*ome they will tell you they will go when they get the time. Later, when you do follow-ups, you will find that they never went there. It became a challenge to me when you do follow up to check if the client goes when you refer, and you will find out that clients didn’t go is stressful. It is very painful to help some clients who are unhelpful, while other are grateful with the services that you are offering. [Participant #8]*
*Theme 3: Managing the socio-economic difficulties faced by their clients*	*Subtheme 3.1. Unemployment among clients and their family members*	*Majority of the clients are unemployed. Sometimes they are unable to look for a job*. *I feel pain when I see clients suffering. I seek donation from the community members to donate what they have. [Participant #4]*
		*Yes, many of our clients are not working. Some of them, they lost their job because of the illness. Because you will see that other clients are in stage that they can’t do anything for themselves and family members. It becomes a challenge, because we don’t have enough resources to fulfil the needs of the clients and family members. [Participant #6]*
		*It’s become difficult, because you find out that some of the clients, they don’t have income at all at home. [*Participant #7]
		*To work with PLWHIV is also a challenge, because many of them are unemployed. Most of them are unable to look for job because of illness. It’s become a challenge, because the clients are not able to fulfil their needs. I feel frustrate, because it will be difficult to meet some needs of the clients, for instance, transport to go to clinic for check-up, because at the organisation, we don’t have transport. Some of the clients we refer them to other [Participant #1]*
		*Yes, many of our clients and family members are not working.’***[Participant #2]**
	*Subtheme 3.2: Lack of food and nutritional support*	*It is bothering, because PLWHIV need food every day so that they can able to take pills or medication. We refer some of them to other organisation[s] to access nutritional support. [Participant #7]*
		*Yes, many of them depended on the social grants and our organisation as we issue food parcels on the monthly basis. It becomes a challenging, because the organisation is difficult to meet some basic need[s] of the clients, because we don’t have enough budget. [Participant #8]*
		*It affects our work, because we don’t have enough nutritional support to assist them. Yes, because some of the client’s needs nutritional support. Some of them, we refer to other organisation[s] to receive food parcels. [Participant #9]*
		*When I do assessment, sometimes I found that at home, no food and no sources of income. When I ask the client, who buy food for you, they respond that they ask next door, or they do door to door asking for food. It’s difficult for me, because at this moment, we don’t have a food parcels at the office and is hard to me to render services to clients who are hungry. [Participant #12]*
		‘*Many clients don’t have a food*. *When I’m incapable of helping clients and family members with food parcel as soon as possible. As a social worker, I feel powerless to not being able to satisfy the needs of the clients. Yes, because there is nothing I can do to offer them food immediately.’* **[Participant #11]**
		‘*We don’t have enough nutritional support for PLWHIV. They depended on the social grant and food that we distribute to them. It becomes a challenge, because we are unable to fulfil the needs of the clients. It’s painful for see clients and their family members suffering.’* **[Participant #3]**
		‘*Shortage of food parcels is distressing, because other clients are not able to work, they depended on the food parcels we distribute to them. I assess the clients’ health status, caregivers’ support, financial status and home and community environment. When I do assessment, sometimes I found that at home, no food and no sources of income. When I ask the client who buy food for you, they respond that they ask next door or they do door to door asking for food. It’s difficult for me, because at this moment, we don’t have a food parcels at the office and is hard to me to render services to clients who are hungry.’* **[Participant #6]**
		‘*Some of the clients and family members, they don’t have any sources of income, so we give them food parcels. It’s become difficult when you find out at the organisation there is a delay of food parcel*. *Yes, some of the clients depend on the food parcels that we give them. It’s painful when the organisation is unable to issue food parcels, when you think that they need food parcels, but you are unable to give them.’* **[Participant 10]**
		‘*It become a challenge, because if you don’t have enough resources like nutritional support to help some of the clients, it became a problem. It is depressing as a social worker not able to help some clients. As social worker should accept that at the organisation that we are working on have a lack of the resources.’* **[Participant #4]**
		‘*Yes, it becomes a challenge, because at the organisation sometimes we struggle to provide nutrition support due to the high numbers of the clients. We distribute food parcels for 200 households every month.* It *becomes a challenge because we have more than 200 households that we have at our organisation. We are not able to help some of the clients with nutritional support. It is painful to see some of clients and family members with hungry stomach.’* **[Participant 13]**
*Theme 4: Challenges associated with resources to render services*	*Subtheme 4.1: Lack of transport for clients to access services*	* … and another member leave support group due to the lack of transport. It’s hard, every time I receive the apologies. Members are often off due to the lack of transport and refreshment.* [Participant #12]
		*In this organisation, we have support group for PLWHIV to support member who feel lonely after HIV diagnosis. I worried, because we don’t have enough resources to run support group, for example, some of them, they want organisation to provide them with transport to attend the session, because they stay far from the organisation. It is very hard to provide them with transport, because we have one car. Eish, so far we recruit member who stay near to the organisation; however, we still looking for a centre venue that will accommodate everyone.* [Participant #3]
		*You will find that the resources that client needs are far from where the client stay, and client don’t have money for transport. [Participant #12]*
		*Yes, sometimes you will find that clients request transport to go to clinic to collect medication. Eish, at the organisation we don’t have enough transport to assist clients. Eish, it becomes a challenge when you find that we are unable to help some clients with transport. [Participant #6]*
		‘*Yes, we check what kind of the resources they need after we refer them, for example, client needs services that we don’t render at the organisation. Sometime is hard to refer clients to community resources. You will find that the resources that client needs are far from where the client stay and client don’t have money for transport.’* **[Participant #8]**
	*Theme 4.2: Inaccessible venues for social workers to render services*	*‘Yes, sometimes it’s difficult to educate community members about HIV. Sometimes it becomes a challenge to hire venue. They need management fees to hire a venue, for example, like community hall. We don’t have enough budget to hire venue*. *I feel stranded not getting a venue, because I don’t know what to do. Sometimes we do door to door; however, is still challenging. I can say it is difficult to deal with some of the cases.’ [Participant #11]*
*Theme 5: Challenges pertaining to community-based interventions*	*Subtheme 5.1: Lack of interest in CHBC services*	*We also do awareness campaign to prevent transmission of the HIV, because some of the community members not welcome us. I can say that it is distressing to do awareness campaign. Some of the community members see us they close the gate, other saying we are tired of you, is better to give us a job. We still doing awareness campaign; however, is discouraging when you know that you are going to face several challenges. [Participant #13]*
		*Yes, especially on World AIDS Day, we do awareness campaign. Unwelcomed by some of the community members. Community members don’t welcome us when we do door-to-door awareness campaign. They also undermine the information that we share with them. Yes, it is very difficult to educate some of them. [Participant #11]*
		*It becomes a problem when some of community members are not interested in instance they will tell you that we are tired to hear HIV every day. [Participant #1]*
		*Yes, we* … *It becomes a challenge when you give them a pamphlet, they throw it away immediately after you give them. It’s frustrating. However, I have to understand the behaviour of the community members, because when you prepared yourself to educate community members and find out they are not interested, nothing can be done. We also continue to educate those who are concerned by inviting them when we have a programme. [Participant #3]*
		‘*Yes, we also do awareness campaign to prevent transmission of the HIV, because some of the community members not welcome us. I can say that it is distressing to do awareness campaign. Some of the community members see us they close the gate, other saying we are tired of you, is better to give us a job. We still doing awareness campaign; however, is discouraging when you know that you are going to face several challenges.’* **[Participant #6]**
		‘*It disappointing to find that some of the community members are not concerned to get new information about HIV because statistics of HIV raise every day. Because when you hear that number of HIV raise, you think about your work, so yes, I am concerned. I can say it is difficult to do community work, especially when you do door to door.’* **[Participant #5]**
		‘*It is difficult indeed. I remember one day we were doing door-to-door awareness campaign, some of the community members when give them pamphlet for HIV, they just cut in small pieces. I really felt discouraged to educate community members about HIV. It’s very difficult to educate someone, more particular someone who does want to be educated.’* **[Participant #10]**
	*Subtheme 5.2: Stigma and discrimination towards social workers, clients and family members*	*As social worker working with PLWHIV, we face stigma and discrimination from the community members. Community member tend to believe that social workers working with PLWHIV are also positive. [Participant #12]*
		*Yes*, *as social workers working with PLWHIV, we also face stigmatisation and discrimination. Once the community members find out that you work with PLWHIV, they try label us and calling me with names … . [Participant #8]*
		*It becomes a challenge when the clients and the family members face discrimination in the community*. *Yes, discrimination still exist in the community. It affects me emotionally, because clients develop negative self-image.* [Participant #10]
		‘*I do family crisis intervention, like client want to disclose the status from the family members, Other client fears to disclose to their family member because they fear of stigma and discrimination therefor, they seek our assistance.*
		‘*Social workers working in the organisation experience stigma and discrimination because the staff member judges us according to our salary’.*
		‘*Social worker who are working in HBC organisation are not receiving the same salary with the social workers working under government, we face stigma according to our salary, we need frequent training for HV/AIDS and social workers working at the organisation are few’.*
		‘*Some families, is hard to accept that one of the family members is positive, they feel disappointed and fear what the community members might say’.*

## Discussion

Community home-based care services are accompanied by various challenges placing social workers in a difficult position. With PLWHIV at the centre of their *microsystem*s, their mutual relations with CHBC organisations through social work services are essential for the functioning of this system. The manifestation of psychosocial difficulties through prolonged disclosure, denial of HIV positive status and lack of familial support signals dysfunctionality of the microsystem, with the potential to spill over into other systems because a dysfunctionality in one system may extend to other systems and impose further complications. In Naick et al. (2018, p. 726) for instance, PLWHIV were reluctant to disclose their HIV-positive statuses, with one indicating the difficulties associated with her preference to disclose to a grandmother while other indicating the need for the right time to do so. Those who did disclose reported negative and discouraging reactions while others generally feared such negative reactions (Naick et al., 2018, p. 727). In Viphonephom et al. (2021, p. 120), caregivers were reportedly worried about the social effect of disclosure particularly the stigma and discrimination which were prominent in the families and communities. In Naick (2013, p. 2), disclosure hampered the linkages of clients to care and treatment and decreased morbidity, mortality, life expectancy and quality of life. Linked to prolonged disclosure is denial of a HIV-positive status which is defined as a ‘defence against anxious forces’ (Kamen et al., 2012, p. 1328).

Denial provide short term benefits and long-term negative consequences because individuals who adopt it benefit immediate relief by evading problems such as stigma while compromising the long-term support benefits associated with disclosure. Although denial may have immediate benefits to clients, it remains an obstacle for social workers because it prevents them from assisting PLWHIV to accept their statuses by delaying therapy (Kulu, 2014, p. 89). Prolonged disclosure and denial can also be linked to the PLWHIV’s ability to seek and receive support, particularly family support, food and nutritional support. An observation by George and McGrath (2019 , p. 876) for instance is that the prolonged or non-disclosure and non-adherence to treatment can be due to HIV-related stigma within the households. In a Brazil, a study of the role of family support networks for PLWHIV by Da Silva and Tavares (2015, p. 114) revealed a failure by family members to provide support to PLWHIV due to various limitations or difficulties. In Woldie et al. (2015, p. 101), nutritional support was one of the challenges faced by PLWHIV, who reported weight loss, worsening immunity leading to non-adherence to Antiretrovirals treatment. In South Africa’s Nelson Mandela Bay, Frood et al. (2018, p. 34) revealed in a study of health and social care professionals’ anguish in rendering care and support to children orphaned by AIDS, appalling and desperate conditions with food parcels taking six months to reach AIDS orphans while social grant took months to process. Considering nutritional challenges from the EST and Farmer’s (1999) perspective it is essential to view it as the manifestation of entrenched poverty and inequality which are perpetuated by historical injustices of the past and which may not easily be resolved through social work interventions. Just like most of the TB patients cited by Framer (2005, p. 149) who are landless peasants living in poverty, so do South African PLWHIV who are mostly cramped in informal settlements where they are unable to produce their own food. These structural factors are clearly beyond the scope of social work practice, hence social workers struggle to provide the necessary support to CHBC programmes.

Despite being essential in supporting HIV programmes, family and nutritional support may not be sufficient on their own. They also require cooperation of PLWHIV with directives and counsel provided by social workers, caregivers and other healthcare professionals and this was proven to be a challenge in this study. Although referral of PLWHIV by social workers and caregivers to other service centres like hospitals or clinics is common, it becomes challenging for social workers when they are ignored. Like the preceding challenges, poor uptake of referrals and refusal of counseling and lack of interest from members of the community should be understood from a broader context of communities that are characterised by poverty, unemployment, stigmatisation and limited or lack of resources (Lamore et al., 2017, p. 2; Oguntibeju et al., 2011, p. 3167; Serena, 2008, p. 100). A generally high level of denial is common among community members, who still consider hospitals to be places to access health care while some prefer to consult traditional healers for treatment instead of seeking healthcare service from the CHBC (Oguntibeju et al., 2011, p. 3167). In Pindani et al. (2013, p. 4), one of the PLWHIV reported the difficulties upholding referrals on time because of transport issues which ultimately result in them taking about a week to access hospitals and clinics to which they were referred. In Woldie et al. (2015, p. 100) Ethiopian study of the needs of PLWHIV in CHBC, Participants reported the significance of economic support among PLWHIV particularly among widows and orphans, who needed shelter and employment security.

Connected to low socio-economic challenges such as unemployment was stigma which became one of their hindrances for securing employment (Kulu, 2014, p. 90; Perri et al., 2021, p. 3). A 2016 study of courtesy stigma among caregivers of people living with HIV pointed to negative attitudes by some community members towards caregivers and PLWHIV. In this study, caregivers reported unwelcoming and negative reactions by community members (Lekganyane, 2016). The challenges reported through this study have clearly mapped Framer’s (2005) interpretation of the causes of poor outcomes of health interventions, classified according to the cognitivist-personalistic involving individual patients which are those that involve the patient individually (i.e. patient’s failure to take treatment as prescribed) and the structural ones which are more inclined to the patient’s poverty conditions (not taking treatment because there is no food since no one is employed in the household/ there is no land to cultivate and grow vegetables and so on).

For social workers to effectively dismantle some of the above challenges, they need resources like transport, venues, and the general support of the community. This is despite CHBC having been envisaged to overcome barriers such as transport costs and waiting periods (Wood et al., 2018, p. 2). Existing research studies revealed that despite being a significant resource for CHBC, transport is a common problem among CHBC organisations (Bateganya et al., 2015; Chang et al., 2013, p. 66; Pindani et al., 2013, p. 1; Woldie et al., 2015, p. 106). These kinds of challenges burden social workers whom instead of focusing on supporting PLWHIV and their patients, spend time addressing these issues. Social workers would need venues such as big halls and schools where they perform activities such as community education and support groups and lack thereof may have dire consequences for CHBC programmes. A South African study of HIV intervention in high schools by Serena (2008, p. 100) revealed how the programmes was conducted in a big school hall where people could not hear presenters while some did not have chairs, with some audience sitting on a floor and tables. Furthermore, one of the schools did not have a hall to facilitate sessions (Serena, 2008, p. 100). These challenges had dire consequences for the success of the programme particularly its envisaged aims. In another South African study of the social construction of identity in HIV home-based care volunteers by Naidu et al. (2012, p. 115) in the KZN, a total population of 12 285 people were served by only one clinic which was rendering family planning and primary healthcare services as well as two community halls and four schools. The success of HIV programmes would be compromised in these situations particularly if social workers were to plan a massive community education programme in the area. Above all, the cooperation of community members, PLWHIV and their family members is essential for the success of CHBC programmes and as demonstrated in this study, such cooperation is a challenge because of lack of interest in CHBC programmes by members of the community. Although lack of cooperation among the community members was common amongst out Participants, it was contrary to Woldie et al. (2015, p. 102) Ethiopian study where community members were actively involved in CHBC by, identifying potential clients and linking them with resourceful organisations in the community.

## Conclusion

Rendering services to PLWHIV through CHBC programme takes a toll on social workers. As demonstrated by our study, social workers working with PLWHIV in CHBC do so under exposure to difficulties such as dealing with who are confronted by psychosocial challenges such as prolonged disclosure, denial of HIV positive status and lack of familial support. In further complicating their duties is a lack of cooperation from PLWHIV themselves who would refuse counseling and not heed to advises for referrals. Drawing from the EST, these challenges are best understood from a broader reflection of the various societal systems in which PLWHIV find themselves and the CHBC programmes are implemented as characterised by stigma and discrimination, dire socio-economic conditions like unemployment and poverty among PLWHIV and their families as well as the overall lack of resources and facilities to efficiently conduct these programmes. Compounding these challenges further, was a lack of interest in CHBC programmes among the community members. This becomes a blow for all the great works that social workers do because without a buy-in from the community, they would not have clients. This situation poses a threat to the success of CHBC programmes because social workers often find themselves resolving the challenges as opposed to implementing their envisaged mandate of the CHBC.

## Recommendations

In view of the findings and conclusions, the following recommendations are proposed:

- Continuous professional Development (CPD) workshops should be designed specifically for social workers in the field of CHBC with the purpose of equipping them with relevant skills to effectively manage clients with psychosocial challenges impacting on the programme;
- Programmes for eliminating HIV-related stigma and encouraging involvement of all community members in CHBC for PLWHIV should be developed and implemented in all affected communities;
- Resources should be mobilised through advocacy programmes to promote funding and provision of resources for the purpose of supporting social workers working with PLWHIV in CHBC programmes;
- Income generating projects should be introduced in CHBC for the benefit of PLWHIV to mitigate the socio-economic challenges impacting on their compliance to treatment and to enable them earn income.

## Data Availability

The authors wish to confirm that data from which the manuscript is based is available within this article and may on request, be availed.

## References

[CIT0001] Bateganya, M. H., Amanyeiwe, U., Roxo, U., & Dong, M. (2015). Impact of support groups for people living with HIV on clinical outcomes: A systematic review of the literature. *Journal of Acquired Immune Deficiency Syndrome*, *15*(68), s368–s374. 10.1097/QAI.0000000000000519 PMC4709521 25768876

[CIT0002] Bester, N., & Herbst, A. (2010). The role of hospice caregivers in caring for families infected with, or affected by, HIV/AIDS. *Social Work/Maatskaplike Werk*, *46(4)*, 450–468. 10.15270/46-4-152

[CIT0003] Campbell, I. D., Campbell, A. R., & Chela, C. (2019). *A community development approach to HIV care, prevention and control*. https://oxfordmedicine.com/view/10.1093med/9780198806653.001.0001/med-9780198806653-chapter-20?print = pdf

[CIT0004] Chang, L. W., Serwadda, D., Quinn, T. C., Wawer, M. J., Gray, R. H., & Reynolds, S. J. (2013). Combination implementation for HIV prevention: Moving from clinical trial evidence to population-level effects. *Lancet Infectious Disease*, *13*(1), 65–76. 10.1016/S1473-3099(12)70273-6 PMC3792852 23257232

[CIT0005] Crawford, M. (2020). Ecological systems theory: Exploring the development of the theoretical framework as conceived by Bronfenbrenner. *Journal of Public Health Issues and Practice*, *4*(2), 170. 10.33790/jphip1100170

[CIT0006] Da Silva, L. M. S., & Tavares, J. S. C. (2015). The family role as a support network for people living with HIV/AIDS a review of Brazilian research into the theme. *Ciência & Saúde Coletiva*, *20*(4), 1109–1118. 10.1590/1413-81232015204.17932013 25923622

[CIT0007] Drakenberg, M., & Malmgren, T. V. (2013). School principal’s perception of 'basic values' in the Swedish compulsory school system regard Bronfenbrenners ecological systems theory. *Social and Economic Education*, *12*(2), 118–128.

[CIT0008] Farmer, P. (1999). *Infections and inequalities: The modern plagues* (1st ed.). University of California Press.

[CIT0009] Framer, P. (2005). *Pathologies of power, health, human rights and the new war on the poor*. University of California Press.

[CIT0010] Freeman, R. J. (2017). *Social workers’ perceptions of their role in providing palliative care to patients with life-limiting illnesses: A qualitative study among social workers in primary care settings in Namibia. Phi (SC) dissertation*. University of South Africa.

[CIT0011] Frood, S., van Rooyen, D., & Ricks, E. (2018). Health and social care professionals’ anguish in providing care and support to children who are AIDS orphans in Nelson Mandela Bay. A qualitative study. *International Journal of Africa Nursing Sciences*, *9*(2018), 31–37. 10.1016/j.ijans.2018.07.002

[CIT0012] George, S., & Mcgrath, N. (2019). Social support, disclosure and stigma and the association with non-adherence in the six months after antiretrovial therapy initiation among a cohort of HIV-positive adults in rural. *AIDS Care*, (7), 875–884. 10.1080/09540121.2018.1549720 30472889 PMC6518453

[CIT0013] Kamen, C., Taniguchi, S., Student, A., Kienitz, E., Giles, K., Khan, C., Lee, S., Gore-Felton, C., & Koopman, C. (2012). The impact of denial on health-related quality of life in patients with HIV. *Quality of Life Research*, *21*(8), 1327–1336. 10.1007/s11136-011-0045-y 22038393

[CIT0014] Kulu, J. A. N. (2014). Social worker’s perspectives on social support needed by people living with HIV/AIDS [MA (SW) dissertation]. Stellenbosch University, Western Cape. https://scholar.sun.ac.za/server/api/core/bitstreams/89ddf514-7079-42d6-aaaf-0268e8bbdb6d/content

[CIT0015] Kupa, P. M., & Geyer, L. S. (2020). A qualitative evaluation of a stress management programme for HIV and AIDS home-based care workers in Tshwane, South Africa. *Journal of Social Aspects of HIV/AIDS*, *17*(1), 1–15. 10.1080/17290376.2020.1810747 PMC7534294 32921228

[CIT0016] Lamore, T., Tilahun, M., & Godana, W. (2017). Determinants for refusal of provider initiated HIV testing and counseling among adult OPD clients in Kachabira district health centers, south Ethiopia: Institution based unmatched case control study. *Health Systems and Policy Research*, *4*. 10.21767/2254-9137.100072

[CIT0017] Larki, M., & Roudsari, R. L. (2019). Home-based care, the missing link in caring of patients living with HIV/AIDS and their family members: A narrative review. *IJCBNM*, *8*(3), 190–208. 10.30476IJCBNM.2020.82771.1085 10.30476/ijcbnm.2020.82771.1085 PMC7334750 32656272

[CIT0018] Lekganyane, M. R. (2016). HIV and AIDS-related courtesy stigma: South African caregivers; experiences and coping strategies. *Alternation*, *23*(2), 120–140. https://portal.issn.org/resource/ISSN/1023-1757

[CIT0019] Lekganyane, M. R. (2017). Experiencing and managing work-related challenges by home-based caregivers caring for people living with HIV and AIDS: Guidelines for support from a social work perspective. DPhil thesis, University of South Africa. https://uir.unisa.ac.za/bitstream/handle/10500/22588/thesis_lekganyane_mr.pdf?sequence=1

[CIT0020] Lekganyane, M. R., & Alpaslan, A. H. (2019). Suggestions by home-based caregivers caring for people living with HIV and AIDS on how social workers could support them in managing their work-related challenges. *Social Work/Maatskaplike Werk*, *55*(2), 140–157. 10.15270/55-2-712

[CIT0021] Mahoney, J., & Ettekal, A. (2017). Ecological systems theory. In K. Peppler (Ed.), *The Sage encyclopedia of out-of-school learning*. Thousand Oaks: SAGE Publications Inc.

[CIT0022] Mamukeyani, E. (2021). Difficulties experienced by caregivers of HIV/AIDS orphans: A qualitative study for rural-based caregivers. *Open Access Library Journal*, *8*(6), 1–15. 10.4236/oalib.1106721

[CIT0023] Moremi, M. Z.. (2012). Volunteers stress and coping in HIV/AIDS home-based care [MA (PS) dissertation]. University of South Africa. https://uir.unisa.ac.za/bitstream/handle/10500/7682/dissertation_moremi_m.pdf?sequence=1&isAllowed=y

[CIT0024] Naick, R, Doherty, T, Jackson, D, Tabana, H, Swanevelder, S, Thea, D., Feeley, F. G., & Fox, M. P. (2013). Linkages to care following home-based HIV counselling and testing intervention in rural South Africa. *Journal of International AIDS Society*, *18*(1), 1–9. 10.7448/IAS.18.1.19843 PMC4461747 26058983

[CIT0025] Naick, R., Zembe, W., Adigun, F., Jackson, E., Tabana, H., Jackson, D., Feeley, F., & Doherty, T. (2018). What influences linkages to care after home-based HIV counseling and testing? *AIDS Behaviour*, *22*(3), 722–732. 10.1007/s10461-017-1830-6 PMC5847222 28643242

[CIT0026] Naidu, T., Sliep, Y., & Dageid, W. (2012). The social construction of identity in HIV/AIDS home-based care volunteers in rural KwaZulu-natal, South Africa. *Journal of Social Aspects of HIV/AIDS*, *9*, 113–126. 10.1080/17290376.2012.683585 23237046

[CIT0027] Navarro, J. L., & Tudge, J. R. H. (2022). Technologizing bronfenbrenner: Neo-ecological theory. *Current Psychology*, 1–17. https://link.springer.com/article/10.1007/s12144-022002738-3 10.1007/s12144-022-02738-3 PMC8782219 35095241

[CIT0028] Ntshwarang, P. N., & Malinga-Musamba, T. (2012). Social workers working with HIV and AIDS in health care settings: A case study of Botswana. *Practice*, *24*(5), 287–298. 10.1080/09503153.2012.703648

[CIT0029] Oguntibeju, O. O., Ndalambo, K. T., & Mokgatle-Nthabu, M. (2011). People living with HIV/AIDS and the utilization of home-based care services. *African Journal of Microbiology Research*, *5*(20), 3166–3174. 10.5897/AJMR11.103

[CIT0030] Perri, M., Craig-Neil, A., Gaspar, M., Hunter, C., Kendall, C., Alexander, O., & Pinto, A. D. (2021). Qualitative study of barriers to employment experienced by people living with HIV in Toronto and Ottawa. *International Journal for Equity in Health*, *20*(36), 1–7. 10.1186/s12939-020-01356-4 33446215 PMC7807879

[CIT0031] Pindani, M., Maluwa, A., Nkondo, M., Nyasulu, B. M., & Chilemba, W. (2013). Perception of people living with HIV and AIDS regarding home-based care in Malawi. *Journal of AIDS Clinical Research*, *4*(3), 1–5. 10.4172/2155-6113.1000201

[CIT0032] Qalinge, L. (2011). Community home-based care programme: A marginalised key community resource. *Social Work/Maatskaplike Werk*, *47(1)*, 51–57. 10.15270/47-1-142

[CIT0033] Reckrey, J. M., Gettenberg, G., Ross, H., Kopke, V., Soriano, T., & ^ Ornstein, K. (2014). The critical role of social workers in home based primary care. *Social Work in Health Care*, *53*(4), 330–343. 10.1080/00981389.2014.884041 24717182 PMC4790723

[CIT0034] Serena, V. F.. (2008). An HIV prevention intervention among high school learners in South Africa [Doctoral dissertation]. University of KwaZulu-Natal, Durban. https://researchspace.ukzn.ac.za/handle/10413/2373

[CIT0035] Valjee, L., & Van Dyk, A. (2014). Impact of caring for people living with HIV on the psychosocial well-being of palliative caregivers. *Curationis*, *37*(1), 1–13. 10.4102/curationis.v37i1.1201 25686108

[CIT0036] Van Dyk, A. (2012). *HIV/AIDS care counselling: A multidisciplinary approach* (5th ed.). Pearson Education.

[CIT0037] Verma, S. P. (2020). The role of professional social worker in awareness of HIV/AIDS among youth. *Current Research on HIV/AIDS: CRHA-121*. 10.29011/2575-7105.100121

[CIT0038] Viphonephom, P., Petitet, P. H., Bertrand, D., Haik-Wagner, N. E., & Paboribourne, P. (2021). Biomedical science’s embodiments in contemporary laos: Informal caregivers and children living with HIV. *Moussons: recherches en sciences humaines sur l'Asie du Sud-Est*, *38*(2021), 111–135. 10.4000/moussons.8057

[CIT0039] White, F., & Hayes, D. L. (2013). *Developmental psychology from infancy to adulthood*. Palgrave.

[CIT0040] Woldie, M., Sudhakar, M., & Feyissa, G. T. (2015). Community home-based care for people living with HIV: An overview of client needs, actors and services provided in Ethiopia. *Journal of AIDS and HIV Research*, *7*(8), 97–108. 10.5897/JAHR2015.0341

[CIT0041] Wood, M. E., Zani, B., Esterhuizen, T. M., & Young, T. (2018). Nurse led home-based care for people with HIV. *BMC Health Services Research*, *18*(219), 1–19. 10.1186/s12913-018-3002-4 29587719 PMC5870334

